# A cobalt-doped iron oxide nanozyme as a highly active peroxidase for renal tumor catalytic therapy†

**DOI:** 10.1039/c8ra05487h

**Published:** 2019-06-17

**Authors:** Yixuan Wang, Hongjun Li, Lihua Guo, Qi Jiang, Feng Liu

**Affiliations:** Department of Nephrology, China-Japan Union Hospital of Jilin University Changchun 130033 China; The Examination Center, China-Japan Union Hospital of Jilin University Changchun 130033 China lihongjun1960@126.com

## Abstract

The Fe_3_O_4_ nanozyme, the first reported nanozyme with intrinsic peroxidase-like activity, has been successfully employed for various diagnostic applications. However, only a few studies have been reported on the therapeutic applications of the Fe_3_O_4_ nanozyme partly due to its low affinity to the substrate H_2_O_2_. Herein, we report a new strategy for improving the peroxidase-like activity and affinity of the Fe_3_O_4_ nanozyme to H_2_O_2_ to generate reactive oxygen species (ROS) for kidney tumor catalytic therapy. We showed that cobalt-doped Fe_3_O_4_ (Co@Fe_3_O_4_) nanozymes possessed stronger peroxidase activity and a 100-fold higher affinity to H_2_O_2_ than the Fe_3_O_4_ nanozymes. The lysosome localization properties of Co@Fe_3_O_4_ enable Co@Fe_3_O_4_ to catalyze the decomposition of H_2_O_2_ at ultralow doses for the generation of ROS bursts to effectively kill human renal tumor cells both *in vitro* and *in vivo*. Moreover, our study provides the first evidence that the Co@Fe_3_O_4_ nanozyme is a powerful nanozyme for the generation of ROS bursts upon the addition of H_2_O_2_ at ultralow doses, presenting a potential novel avenue for tumor nanozyme catalytic therapy.

## Abstract 2

Cobalt dopant in Fe_3_O_4_ nanozymes improved their binding affinity to H_2_O_2_ and enhanced the tumor catalytic therapy efficacy.

## Graphical abstract

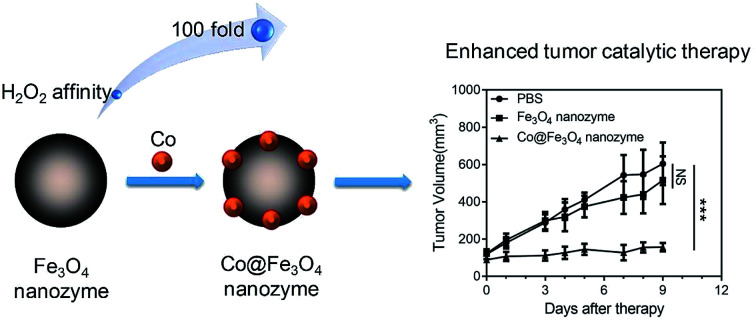

## Introduction

Nanozymes are a class of nanomaterials with intrinsic enzyme-like activities.^1–3^ Over the last decade, a wide variety of nanomaterials have been reported to possess natural enzyme-like activities.^1–5^ The biochemical reactions catalyzed by these types of nanozymes exhibit similar enzymatic kinetics as in the case of natural enzymes. Nanozymes exhibit comparable enzymatic activity but with much higher stability and lower cost as compared to natural enzymes. In addition, their activities are tunable, and they can be easily integrated with nanosystems to achieve multifunctionality;^6,7^ therefore, nanozymes possess significant potential for a wide range of applications in biomedicine such as in immunoassays, biosensors, and antibacterial and antibiofilm agents.^4,8,9^

As a classical magnetic nanomaterial, iron oxide (Fe_3_O_4_) nanoparticles are the first reported nanozyme with intrinsic peroxidase-like activity.^10,11^ Fe_3_O_4_ nanozymes with intrinsic magnetic properties have been extensively used for biological applications including magnetic resonance imaging, magnetic drug delivery, magnetic hyperthermia and magnetic separation.^12–14^ Based on its newly discovered catalytic properties, the Fe_3_O_4_ nanozyme can act as a multifunctional enzyme mimetic for versatile biomedical applications.^12^

Recently, significant efforts have been made to explore the feasibility of application of nanozymes in *in vivo* clinical diagnosis and therapy.^9,15–18^ As the first well-studied nanozyme, Fe_3_O_4_ nanozymes have already been evaluated in tumor catalytic therapy for catalyzing the decomposition of hydrogen peroxide to generate ROS.^16,19,20^ However, because of the low affinity of the Fe_3_O_4_ nanozymes to H_2_O_2_, Fe_3_O_4_ nanozyme-based catalytic therapy typically requires an additional high dose of H_2_O_2_ (approximately 10^−3^ to 10^−4^ M);^19,20^ this makes this nanozyme-based catalytic tumor therapy strategy unviable for practical application.

Some heterogeneous oxide nanomaterials, such as ZnFeO_3_^21^ and NiFeO_4_^22^, formed by iron and other metals have been reported to exhibit enhanced peroxidase-like behavior; this indicates that transition metal doping of Fe_3_O_4_ nanozymes may be an effective way to improve the enzymatic activity of these nanoenzymes;^23^ interestingly, Chen *et al.* have reported that Fe–Co bimetallic alloy nanoparticles also exhibit high peroxidase-like activity.^24^ Moreover, Vetr *et al.* have investigated the effect of transition metal (Co, Ni, and Zn) doping on the catalytic performance of Fe_3_O_4_ nanozymes. They have demonstrated that NiFe_2_O_4_ and ZnFe_2_O_4_ NPs exhibit lower catalytic activity as compared to CoFe_2_O_4_ NPs.^25^ Thus, doping of cobalt, a non-noble metal, into Fe_3_O_4_ nanozymes is a promising method to improve the peroxidase-like activity of Fe_3_O_4_ nanozymes; however, all these studies focus on the *in vitro* biosensing applications of metal-doped Fe_3_O_4_ nanozymes, and the applications of these nanozymes in tumor catalytic therapy have not been explored.

In this study, we demonstrated that doping of Co into Fe_3_O_4_ nanozymes (Co@Fe_3_O_4_) resulted in not only excellent peroxidase-like activity, but also a 100-fold higher affinity of Co@Fe_3_O_4_ to H_2_O_2_ than that in the case of Fe_3_O_4_ nanozymes. By employing Co@Fe_3_O_4_ nanozymes, we successfully achieved effective antitumor activity with the addition of an ultralow dose (10 nM) of H_2_O_2_ both *in vitro* and *in vivo*. This study provides a promising strategy to enhance the peroxidase-like activity of the Fe_3_O_4_ nanozyme and achieves the purpose of Fe_3_O_4_ nanozyme based-renal tumor catalytic therapy.

## Materials and methods

### Materials

Chemicals and materials were supplied by Sigma-Aldrich (St. Louis, MO) unless otherwise specified.

### Synthesis and characterization of the Fe_3_O_4_ and Co@Fe_3_O_4_ nanozymes

The Fe_3_O_4_ nanozymes and Co-doped Fe_3_O_4_ nanozymes were synthesized according to the solvothermal method reported in the literature^10,26^ with some modifications. Briefly, for the Fe_3_O_4_ nanozymes, FeCl_3_·6H_2_O (0.82 g) was dissolved in 40 mL ethylene glycol. When the solution became clear, NaAc (3.6 g) was added under continuous vigorous stirring for 30 min. The mixture was sonicated for 10 min, then transferred to a 50 mL Teflon-lined stainless-steel autoclave and reacted at 200 °C for 12 h. After the reaction was completed, the autoclave was cooled down to room temperature. Then, the products obtained were washed several times with ethanol and dried at 60 °C.

The Co@Fe_3_O_4_ nanozymes were also synthesized using the same procedure but extra Co(NO_3_)_3_·6H_2_O (0.82 g) was added to the reaction system.

The morphology and structure of the Fe_3_O_4_ and Co@Fe_3_O_4_ nanozymes were characterized by transmission electron microscopy (TEM, JEOL JEM-1400 120 kV), scanning electron microscopy (SEM, Zeiss Supra55) and dynamic light scattering (DLS, DynaPro Titan). Energy dispersive X-ray spectroscopy (EDX) of the Fe_3_O_4_ and Co@Fe_3_O_4_ nanozymes was conducted using the Tecnai G2 F30 instrument. X-ray diffraction (XRD) measurements were performed using the X'Pert pro Philips X-ray powder diffractometer. X-ray photoelectron spectroscopy (XPS) was performed by the ESCALab220i-XL high-performance electron spectrometer with a monochromatic Al Kα source.

### Kinetic analysis of the Fe_3_O_4_ and Co@Fe_3_O_4_ nanozymes

The kinetic parameters of the Fe_3_O_4_ and Co@Fe_3_O_4_ nanozymes were determined by monitoring the absorbance change at 652 nm using the iMark™ Microplate Reader (Bio-Rad, USA) in the time course mode at room temperature. Kinetic assays were carried out using the Fe_3_O_4_ nanozymes (0.2 μg) or Co@Fe_3_O_4_ nanozymes (0.2 μg) in a 100 μL of reaction buffer (0.2 M NaAc buffer, pH 4.5) in the presence of H_2_O_2_ and TMB. The kinetic analysis of Fe_3_O_4_ and Co@Fe_3_O_4_ with H_2_O_2_ as the substrate was performed by varying the concentrations of H_2_O_2_ with 0.8 mM TMB and *vice versa*. The absorbance (652 nm) changes were calculated relative to the changes in the molar concentration of TMB using the molar absorption coefficient of 39 000 M^−1^ cm^−1^ for the TMB-derived oxidation products according to the Beer–Lambert law.^27^ All the measurements were performed at least in triplicate, and the values were then averaged. The results are provided as mean ± the standard deviation (SD). The Michaelis–Menten constant was calculated using the Lineweaver–Burk plots of the double reciprocal of the Michaelis–Menten equation *ν* = *V*_max_ × [S]/(*K*_M_ + [S]) by GraphPad Prism 6.02 (GraphPad Software), where *ν* is the initial velocity, *V*_max_ is the maximal reaction velocity, [S] is the concentration of the substrate and *K*_M_ is the Michaelis–Menten constant.

### ESR spectroscopy measurements

The ESR measurements were carried out using a Bruker electron spin resonance (ESR) spectrometer (A300-10/12, Germany) at ambient temperature. Herein, fifty microliter aliquots of the control or sample solutions were put in glass capillary tubes with the internal diameters of 1 mm and sealed. The capillary tubes were then inserted into the ESR cavity, and the spectra were obtained at selected times. The instrument settings are as follows: 1 G field modulation, 100 G scan range, and a 20 mW microwave power for the detection of spin adducts using spin traps. The spin trap BMPO was employed to verify the formation of hydroxyl radicals (OH˙) during the degradation of H_2_O_2_ in the presence of the Fe_3_O_4_ or Co@Fe_3_O_4_ nanozymes under the same conditions. The amount of hydroxyl radicals was quantitatively estimated by the ESR signal intensity of the hydroxyl radical spin adduct (BMPO/OH˙) using the peak-to-peak height of the second line of the ESR spectrum.

### Cell viability assay

The cytotoxicity of the Fe_3_O_4_ and Co@Fe_3_O_4_ nanozymes with the addition of 10 nM H_2_O_2_ was determined using the CCK-8 cell viability assay kit (Dojindo Molecular Technologies). Briefly, A-498 cells (Human renal cancer cell, ATCC, HTB-44) were plated in 96-well plates (BD Biosciences) with the density of 5 × 10^3^ cells per well and cultured in 100 μL EMEM (Catalog No. 30-2003) for 1 day before the addition of Fe_3_O_4_,Co@Fe_3_O_4_ nanozymes, or only the buffer as a control. On each plate, blank wells (*n* = 6) with media were defined as 0% viability. Moreover, the wells with only PBS-treated cells (*n* = 6) were defined as 100% viability. The dilutions of the Fe_3_O_4_ and Co@Fe_3_O_4_ nanozymes were prepared using a buffer containing 10 nM H_2_O_2_. The cells were then exposed to the Fe_3_O_4_ or Co@Fe_3_O_4_ nanozymes at a series of concentrations (from 0 to 0.2 mg mL^−1^) for 24 hours. After stimulation, a 10 μL CCK-8 solution was added to each well. The plates were then incubated for 4 h at 37 °C. After this, the absorbance was determined at 450 nm using the Benchmark Plus microplate spectrophotometer (Bio-Rad Laboratories, Inc.). The results presented herein are the average of those obtained *via* three independent experiments.

### Localization of the Fe_3_O_4_ and Co@Fe_3_O_4_ nanozymes in cytoplasm

The cellular uptake and distribution of Fe_3_O_4_ or Co@Fe_3_O_4_ nanozymes in human renal tumor cells were investigated by a confocal laser scanning microscope. Briefly, the A-498 cells were plated on poly-l-lysine-treated coverslips (BD Biosciences) and cultured in a six-well plate (Corning) for 12 h before use. After stimulation for 48 h with the Alexa-488-labeled Fe_3_O_4_ or Co@Fe_3_O_4_ nanozymes (0.2 mg mL^−1^), the cells were washed with PBS, fixed in 4% cold formaldehyde in PBS for 5 min, and then permeabilized with 0.1% Triton X-100. After being washed with PBS, the cells were blocked in a 5% normal goat serum for 30 min at room temperature. To visualize the lysosomes, the cells were incubated with anti-Lamp1 mAb (1 : 200, clone H4A3; Invitrogen) at 37 °C for 1 h. The cells were then washed three times with PBS and incubated with goat anti-mouse IgG1 conjugated with Alexa-555 (1 : 500; Invitrogen) for 1 h at 37 °C. Finally, the nuclei of the cells were stained with 4′,6′-diamidino-2-phenylindole (DAPI, 1 μg mL^−1^, Roche Applied Science) for 10 min at room temperature. The samples were examined using a confocal laser scanning microscope (Olympus FluoView FV-1000, Tokyo, Japan).

### Intracellular ROS assay

The fluorescent probe 2′,7′-dichlorofluorescin diacetate (H_2_DCFDA, Sigma-Aldrich, D6883) was used to measure the intracellular generation of ROS by the Fe_3_O_4_ or Co@Fe_3_O_4_ nanozymes. Briefly, the confluent A-498 cells on the coverslips (BD Biosciences) were incubated with Fe_3_O_4_ or Co@Fe_3_O_4_ nanozymes (0.2 mg mL^−1^) for 4 hours. After being washed with PBS, the cells were incubated with 10 μM H_2_DCFDA in a serum-free DMEM for 20 min at 37 °C in the dark. The fluorescence intensities of H_2_DCFDA were measured by a confocal laser scanning microscope (Olympus FluoView FV-1000, Tokyo, Japan).

### Apoptosis analysis

The apoptosis analysis of the treated tumor cells was conducted by PI and annexin V staining and flow cytometry (FACSCaliburTM, Becton Dickinson, Franklin Lakes, NJ, USA). Briefly, the Fe_3_O_4_ and Co@Fe_3_O_4_ (0.2 mg mL^−1^) nanozymes were incubated with the A-498 tumor cell lines for 24 h. After trypsinization, the treated A-498 tumor cells were incubated with annexin V and PI for 15 min to achieve nuclear staining. After this, the cells were fixed and incubated with streptavidin-fluorescein (5 μg mL^−1^) (Sigma, USA) for 15 min. Cell death was evaluated by the quantification of annexin-stained apoptotic cells and PI-stained necrotic cells using flow cytometry.

### Therapy studies

Herein, eighteen female BALB/c nude mice bearing A-498 tumors were randomly assigned to four groups (*n* = 6 mice per group). All the mice were intratumorally treated with a single dose of Fe_3_O_4_ and Co@Fe_3_O_4_ nanozymes (3 mg mL^−1^, 100 μL) with 10 nM H_2_O_2_ when the diameter of the tumors was about 100 mm^3^. For the controls, PBS was administered. The tumor size was measured 3 times a week. The tumor size was calculated as volume [mm^3^] = length × width^2^ × π/6. The measured values are presented as mean ± SD.

## Results

### Characterization of the Co@Fe_3_O_4_ nanozymes

The Fe_3_O_4_ nanozymes and Co-doped Fe_3_O_4_ nanozymes (Co@Fe_3_O_4_) used in this study were synthesized by the solvothermal method. To study the composition of the as-prepared nanozymes, the EDX analysis was performed. As shown in Fig. S1,† the EDX spectrum of the Co@Fe_3_O_4_ nanozymes indicated that the Fe and Co elements were present in the nanoparticles. Based on the EDX mapping analysis, the content of Fe and Co in the Co@Fe_3_O_4_ nanozymes were determined as 33.48% and 16.23%, respectively (Table S1†). In conclusion, herein, the synthesized Co@Fe_3_O_4_ nanozymes contained Fe and Co with the ratio of approximately 2 : 1; this confirmed that Co was successfully doped into the Fe_3_O_4_ nanozymes by the simple solvothermal method.

**Fig. 1:**
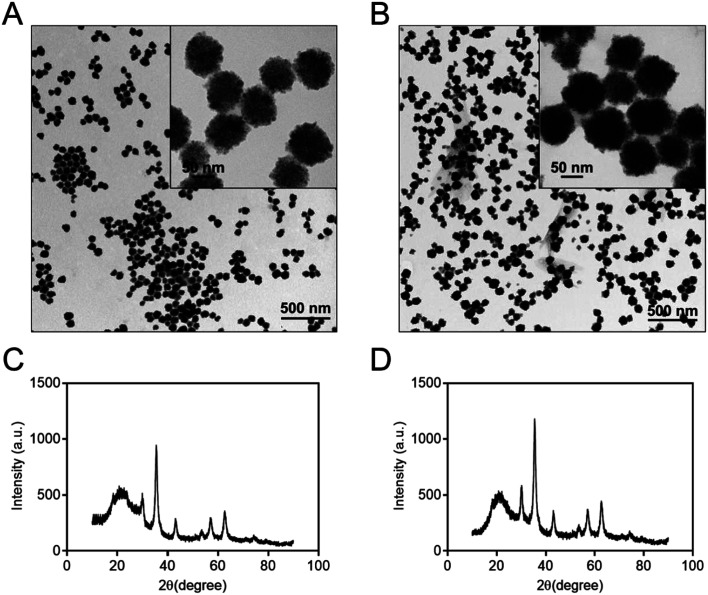
TEM images and XRD diffraction patterns of the Fe_3_O_4_ (A and C) and Co@Fe_3_O_4_ nanozymes (B and D), respectively.

To characterize the structure of the Co@Fe_3_O_4_ nanozymes, TEM, SEM, DLS and X-ray diffraction (XRD) analysis were performed. The TEM images of the as-prepared Fe_3_O_4_ and Co@Fe_3_O_4_ nanozymes are shown in Fig. 1A and B, respectively. The SEM images of the Fe_3_O_4_ and Co@Fe_3_O_4_ nanozymes are presented in Fig. S2A and B,† respectively. The results indicate that the Fe_3_O_4_ and Co@Fe_3_O_4_ nanozymes present a typical spherical morphology. The average size of the Fe_3_O_4_ nanozymes was determined to be 89.8 ± 7.9 nm by the TEM images, whereas that of the Co@Fe_3_O_4_ nanozymes was determined to be 94.6 ± 8.6 nm. Moreover, the Fe_3_O_4_ and Co@Fe_3_O_4_ nanozymes exhibited the average size of 90.31 ± 0.62 nm and 95.82 ± 3.57 nm in solution (Fig. S2C and D†), respectively. The XRD patterns of the as-prepared nanozymes are shown in Fig. 1C and D, which indicate that both the Fe_3_O_4_ and Co@Fe_3_O_4_ nanozymes are well crystallized. Moreover, each characteristic diffraction peak of the Co@Fe_3_O_4_ nanozymes was similar to that of the Fe_3_O_4_ nanozymes and the standard PDF card of Fe_3_O_4_ (JCPDS card no. 19-0629); this indicated that Co-doping of the Fe_3_O_4_ nanozymes did not affect the phase pattern of Fe_3_O_4_.

**Fig. 2:**
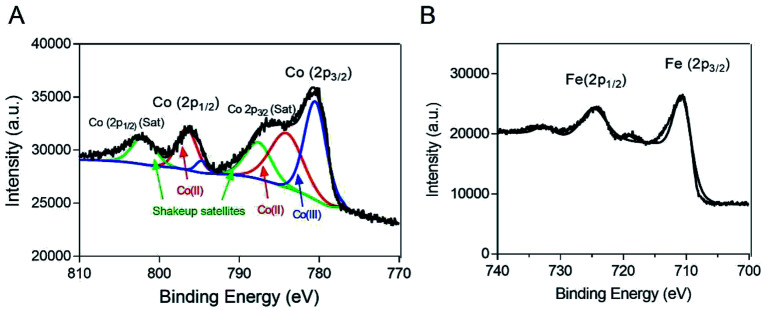
XPS spectra of the Co@Fe_3_O_4_ nanozyme. (A) The Co 2p XPS spectrum of the Co@Fe_3_O_4_ nanozyme. (B) The Fe 2p XPS spectrum of the Co@Fe_3_O_4_ nanozyme.

To characterize the oxidation state of cobalt in the Co@Fe_3_O_4_ nanozyme, we further performed XPS analysis of the as-prepared Co@Fe_3_O_4_ nanozyme. The high-resolution XPS spectrum of Co 2p is shown in Fig. 2A. The Co 2p XPS peak at 780.8 eV was assigned to Co (2p_3/2_), with a shake-up satellite peak at 785.9 eV. In addition, the Co 2p XPS peak at 797.2 eV was attributed to Co (2p_1/2_), with a satellite peak at 803.0 eV.^28^ These characteristic and satellites peaks confirm that Co^2+^ is present in the Co@Fe_3_O_4_ nanozyme. Moreover, as shown in Fig. 2B, the Fe 2p XPS spectrum exhibited characteristic peaks with the binding energy values at 711.0 and 724.0 eV, assigned to the Fe (2p_3/2_) and Fe (2p_1/2_) peaks,^29^ respectively. Since the atomic radius of iron (140 pm) is similar to that of the cobalt atom (135 pm), these results suggest that the cobalt atoms are probably located only at the lattice positions of the Fe_3_O_4_ crystal structure.

### Peroxidase-like activity and steady-state kinetic assay of the Co@Fe_3_O_4_ nanozymes

**Fig. 3:**
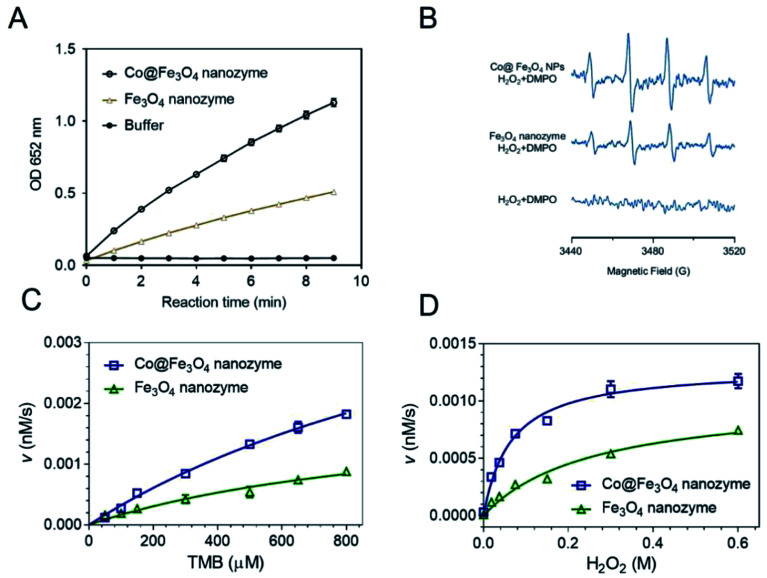
Steady-state kinetic assay and the catalytic mechanism study for the Fe_3_O_4_ and Co@ Fe_3_O_4_ nanozymes. (A) Comparison between the peroxidase-like activities of the Fe_3_O_4_ and Co@Fe_3_O_4_ nanozymes. (B) ESR detection of the hydroxyl radical generation during the catalytic reactions of the Fe_3_O_4_ and the Co@Fe_3_O_4_ nanozymes. (C–D) Kinetic assay of Fe_3_O_4_ and Co@Fe_3_O_4_ nanozymes with TMB (C) or H_2_O_2_ (D). For C, the concentration of H_2_O_2_ was 100 mM, whereas the TMB concentration varied. For (D), the concentration of TMB was 0.8 mM, whereas the H_2_O_2_ concentration varied.

To directly compare the peroxidase-like activity of the Fe_3_O_4_ and Co@Fe_3_O_4_ nanozymes, we performed typical catalytic experiments using the peroxidase substrate 3,3′,5,5′-tetramethylbenzidine (TMB) and H_2_O_2_ as previously reported.^11^ The results showed that both the Fe_3_O_4_ and Co@Fe_3_O_4_ nanozymes catalyzed the oxidation of TMB with H_2_O_2_ to produce blue color products with absorption at 652 nm (Fig. 3A). Moreover, the results demonstrated that the Co@Fe_3_O_4_ nanozymes exhibited a significant improvement in the peroxidase-like activity as compared to the Fe_3_O_4_ nanozymes; this indicated that a significant improvement in the nanozyme activity was achieved by Co doping of the Fe_3_O_4_ nanozymes.

The mechanism of action of the Co@Fe_3_O_4_ nanozymes was investigated using the ESR method. As shown in Fig. 3B, similar to the previously reported Fe_3_O_4_ nanozymes, the Co@Fe_3_O_4_ nanozymes significantly enhanced the generation of hydroxyl radicals under acidic conditions. Importantly, the Co@Fe_3_O_4_ nanozymes generated more hydroxyl radicals than the Fe_3_O_4_ nanozymes under the same conditions; this further confirmed that Co doping significantly improved the peroxidase-like activity of the Fe_3_O_4_ nanozymes.

To obtain the apparent kinetic parameters of the Co@Fe_3_O_4_ nanozymes, the Michaelis–Menten experiments were performed. Fig. 3C and D show the typical kinetics for TMB and H_2_O_2_, respectively. The apparent Michaelis–Menten constant (*K*_M_) and the maximum initial reaction rate (*V*_max_) of the Co@Fe_3_O_4_ and Fe_3_O_4_ nanozymes were calculated. Moreover, these kinetic parameters of the Co@Fe_3_O_4_ nanozymes were compared with those of the Fe_3_O_4_ and Co_3_O_4_ nanozymes and the natural enzyme HRP (Table 1). The Fe_3_O_4_ nanozymes typically exhibited low affinity to H_2_O_2_. The *K*_M_ value to H_2_O_2_ for the Co@Fe_3_O_4_ nanozymes was much lower than that for the Fe_3_O_4_ and Co_3_O_4_ nanozymes; this indicated that there was a significant improvement in the affinity of the nanozymes towards substrates after Co doping. More importantly, the *K*_M_ value to H_2_O_2_ for Co@Fe_3_O_4_ was nearly 50-fold and 100-fold lower than that of the HRP enzyme and the Fe_3_O_4_ nanozymes, respectively; this demonstrated that the Co@Fe_3_O_4_ nanozymes exhibited much higher affinity to H_2_O_2_ than HRP and the other nanozymes. The *V*_max_ values to H_2_O_2_ for the Co@Fe_3_O_4_ nanozymes were also significantly improved.

**Table 1:** Comparison between the apparent Michaelis–Menten constants (*K*_M_) and maximum initial reaction rates (*V*_max_) of the Fe_3_O_4_, Co@Fe_3_O_4_, Co_3_O_4_ nanozymes and horseradish peroxidase (HRP) enzymes

Nanozyme	*K*_M_ (mM)		*V*_max_ (10^−8^ M^−1^ s^−1^)		References
	H_2_O_2_	TMB	H_2_O_2_	TMB	
Co@Fe_3_O_4_	0.19	1.17	71.5	37.9	This work
Fe_3_O_4_	56.89	1.06	59.6	16.8	This work
Co_3_O_4_	1.14	5.09	1.72	9.98	24
HRP	10.35	3.95	0.689	37.65	11

### Anti-tumor activities and mechanistic study of the Co@Fe_3_O_4_ nanozymes

Tumor cells typically possess higher levels of endogenous H_2_O_2_ and reactive oxygen species (ROS) than normal cells.^9,20^ The balance of the ROS determines the fate of the tumor cells. It has been previously shown that stimulation of ROS is a common strategy for cancer chemotherapy.^30,31^ Thus, we employed the Co@Fe_3_O_4_ nanozymes to trigger the burst of ROS to kill the tumor cells.

Fe_3_O_4_ nanozymes, as the first well-studied nanozyme, have already been evaluated in tumor catalytic therapy for catalyzing the decomposition of hydrogen peroxide to generate ROS.^19,20^ However, because of the low affinity of these nanozymes to H_2_O_2_, the Fe_3_O_4_ nanozyme-based catalytic therapy typically requires additional high doses of H_2_O_2_ (approximately 10^−3^ to 10^−4^ M);^19,20^ this makes this nanozyme-based catalytic tumor therapy strategy unfeasible for practical application. In this study, we demonstrated that the Co@Fe_3_O_4_ nanozymes exhibited a 100-fold higher affinity to H_2_O_2_ than the Fe_3_O_4_ nanozymes. Therefore, we next evaluated the catalytic antitumor activity of the Co@Fe_3_O_4_ nanozymes with ultra-low doses of H_2_O_2_.

**Fig. 4:**
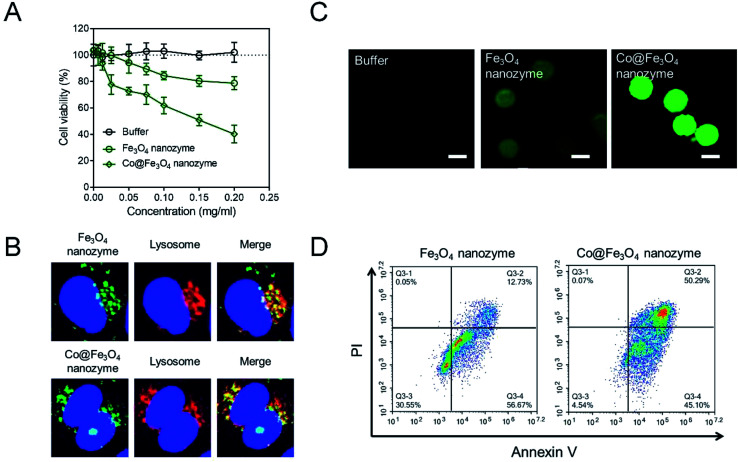
The antitumor cell activities and mechanistic study of the Fe_3_O_4_ and Co@Fe_3_O_4_ nanozymes *in vitro*. (A) Cell viability of the human renal cancer cells A-498 incubated with the Fe_3_O_4_ and Co@Fe_3_O_4_ nanozymes. The buffer contained 10 nM H_2_O_2_. (B) Localization of the Fe_3_O_4_ and Co@Fe_3_O_4_ nanozymes in the A-498 cells. (C) The ROS level in the A-498 cells after stimulation with nanozymes and 10 nM H_2_O_2_. Scale bar = 20 μm. (D) Apoptosis analysis of the A-498 cells after incubation with nanozymes and 10 nM H_2_O_2_.

Considering that the typically used concentration of H_2_O_2_ is around 10^−3^ to 10^−4^ M, we have tried to use 10 nM (10^−8^ M) H_2_O_2_ to evaluate the antitumor activities of the Co@Fe_3_O_4_ nanozymes. As shown in Fig. 4A, the buffer group containing 10 nM H_2_O_2_ exhibited no significant toxicity to kidney cancer cells; this indicated that the tumor cells were able to survive at 10 nM H_2_O_2_. After incubation with 0.2 mg mL^−1^ Fe_3_O_4_ nanozymes and 10 nM H_2_O_2_ for 24 hours, only less than 20% tumor cells were killed. These results are consistent with the previously reported results. Only a high dose of H_2_O_2_ allows the Fe_3_O_4_ nanozymes to effectively kill tumor cells. In the case of the Co@Fe_3_O_4_ nanozymes, 0.02 mg mL^−1^ Co@Fe_3_O_4_ nanozymes with 10 nM H_2_O_2_ achieved similar antitumor activities as 0.2 mg mL^−1^ Fe_3_O_4_ nanozymes. Moreover, 0.2 mg mL^−1^ Co@Fe_3_O_4_ nanozymes and 10 nM H_2_O_2_ killed more than 60% of the tumor cells within 24 hours. Thus, the Co@Fe_3_O_4_ nanozymes effectively killed tumor cells with the addition of H_2_O_2_ at ultralow doses.

As is well-known, the Fe_3_O_4_ nanozymes exhibit peroxidase-like activity only under acidic conditions.^12^ Since the Co@Fe_3_O_4_ nanozymes exhibit significant antitumor activity, we infer that the Co@Fe_3_O_4_ nanozymes localize in the lysosome (pH 4–5) after incubation with the tumor cells. To verify this hypothesis, we labeled the nanozymes with Alexa Fluor 488 to track their intracellular localization. As shown in Fig. 4B, we found that after incubation with tumor cells for 4 hours, most of the internalized Fe_3_O_4_ nanozymes co-localized with lysosomes. Similar to the Fe_3_O_4_ nanozymes, nearly all of the internalized Co@Fe_3_O_4_ nanozymes localized in the lysosomes, the highly acidic microenvironment of which would favor the peroxidase-like activities. Thus, the co-localization analysis of the nanozymes and lysosomes demonstrated that the nanozyme-based tumor catalytic therapy strategy is feasible.

In our hypothesis, the antitumor activities of the Co@Fe_3_O_4_ nanozymes are attributed to the catalytic generation of ROS by the decomposition of hydrogen peroxide, resulting in oxidative stress in the tumor cells. To verify this hypothesis, the intracellular ROS levels in the tumor cells were detected by employing 2′,7′-dichlorofluorescein diacetate (H_2_DCFDA), a typical ROS fluorescent dye. As shown in Fig. 4C, the tumor cells treated with only 10 nM H_2_O_2_ exhibited no significant ROS signal. After incubation with the Fe_3_O_4_ nanozymes and 10 nM H_2_O_2_, the green fluorescence intensity increased. In contrast, the tumor cells treated with the Co@Fe_3_O_4_ nanozymes and 10 nM H_2_O_2_ presented strong green fluorescence intensity, indicating that the Co@Fe_3_O_4_ nanozymes catalyzed the decomposition of H_2_O_2_ to generate an ROS burst to cause cell apoptosis. As shown in Fig. 4D, the tumor cells treated with the Co@Fe_3_O_4_ nanozymes and 10 nM H_2_O_2_ exhibited a significant apoptosis pattern. When the tumor cells were stimulated with the nanozymes at same concentration, the apoptosis induced by the Co@Fe_3_O_4_ nanozymes in the tumor cells was 4-fold higher than that of the Fe_3_O_4_ nanozymes.

**Fig. 5:**
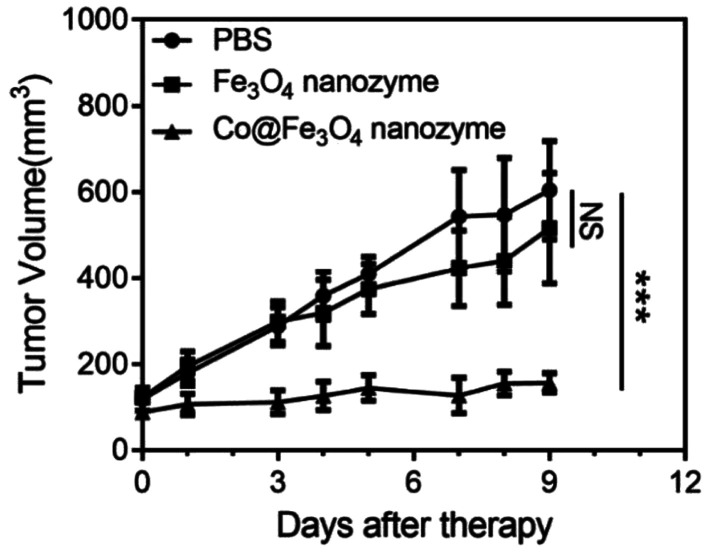
Antitumor activities of the Fe_3_O_4_ and Co@Fe_3_O_4_ nanozymes *in vivo*. *n* = 6, ***p < 0.001, NS, no significance, unpaired Student's *t* test on day 9.

To further evaluate the antitumor activity of the Co@Fe_3_O_4_ nanozymes *in vivo*, we employed the human renal cancer cell A-498 xenograft in nude mice as a tumor model. The Fe_3_O_4_ nanozymes and Co@Fe_3_O_4_ nanozymes were intratumorally injected at the dose of 0.3 mg in 100 μL PBS and 10 nM H_2_O_2_ when the tumor volume reached 100 mm^3^. After this, the tumor volumes were determined 3 times a week. As shown in Fig. 5, the Co@Fe_3_O_4_ nanozyme-treated mice exhibited significant tumor inhibition after Co@Fe_3_O_4_ administration, whereas the Fe_3_O_4_ nanozyme-treated mice exhibited only slight tumor inhibition when compared with the PBS-treated mice. Thus, the Co@Fe_3_O_4_ nanozymes exhibited excellent *in vivo* renal tumor catalytic therapy activity, whereas the Fe_3_O_4_ nanozymes only partially inhibited the renal tumor growth due to their relative low peroxidase activity and low binding affinity to H_2_O_2_;^11^ this was consistent with previous studies.^9^

Overall, these results provide strong evidence that the Co@Fe_3_O_4_ nanozymes possess the ability to regulate intracellular ROS upon the addition of H_2_O_2_ at ultralow concentrations. Once located in the acidic microenvironment of lysosomes, these nanozymes induce cell death by boosting the level of ROS. The Co@Fe_3_O_4_ nanozymes exhibited significant antitumor activities against human renal tumor both *in vitro* and *in vivo*.

## Discussion and conclusion

ROS-induced apoptosis is a popular strategy for cancer therapy.^32–34^ The tumor therapy strategies utilizing nanozymes mainly act by stimulating the production of ROS.^9^ The Fe_3_O_4_ nanozymes can simulate peroxidase and thereby efficiently catalyze the decomposition of H_2_O_2_ to generate ROS to inhibit tumors *in vivo*. However, the low binding affinity of the Fe_3_O_4_ nanozyme to H_2_O_2_ and its relatively low catalytic activity limit the development of the Fe_3_O_4_ nanozyme-based tumor catalytic therapy.

Transition metal doping has been demonstrated to be an effective and easy way to improve the peroxidase-like activity of Fe_3_O_4_ nanozymes.^23^ Among the transition metals, cobalt, a non-noble metal, has been proven to be a promising dopant to enhance the enzymatic activity of the Fe_3_O_4_ nanozyme.^25^ Importantly, Chen *et al.* have systematically studied the effects of doping Fe/Co at different ratios on the enzymatic activity of the Fe_3_O_4_ nanozyme. They have demonstrated that when the ratio of Fe/Co is around 2 : 1, the peroxidase-like activity of the Co-doped Fe_3_O_4_ nanozyme is the best enzymatic activity.^24^ In this study, by employing a simple solvothermal method, we fabricated the Co@Fe_3_O_4_ nanozyme with the ratio of Fe/Co around 2 : 1. Compared with the case of other strategies, including metal doping, biomimetic coating, and C-dot modification methods, that significantly improved the peroxidase-like activity of the Fe_3_O_4_ nanozyme, our Co@Fe_3_O_4_ nanozyme exhibited the best binding affinity to H_2_O_2_ (Table S2†).

The XPS and EDX analysis of the Co@Fe_3_O_4_ nanozyme demonstrated that the cobalt atoms were probably located only at the lattice positions of the Fe_3_O_4_ crystal structure. Although the Co atom possesses a similar size as the Fe atom, the Co atoms doped into the Fe_3_O_4_ crystal may still slightly change the surface physical environment,^35^ resulting in an improved binding affinity of the nanozyme to H_2_O_2_. In addition, the Co dopant may produce more catalytically active sites and substrate-binding sites on the surface of the Co@Fe_3_O_4_ nanozyme when compared with the case of the Fe_3_O_4_ nanozyme.^36^ Moreover, the higher redox potential of Co^3+^/Co^2+^ (1.30 V) as compared to that of Fe^3+^/Fe^2+^ (0.771 V) in the Fe_3_O_4_ nanozyme may be another reason for the improvement in the peroxidase-like activities of Co@Fe_3_O_4_.^37,38^

In conclusion, using a simple solvothermal method, we successfully synthesized Co-doped Fe_3_O_4_ (Co@Fe_3_O_4_) nanozymes that contained Fe and Co at the ratio of approximately 2 : 1. The well-crystallized Co@Fe_3_O_4_ nanozymes exhibited excellent peroxidase-like activity. More importantly, Co doping makes the Co@Fe_3_O_4_ nanozymes exhibit a 50-fold and 100-fold higher affinity to H_2_O_2_ than that of the HRP and Fe_3_O_4_ nanozymes, respectively. The improvement of the H_2_O_2_ affinity renders the Co@Fe_3_O_4_ nanozymes with excellent antitumor activity upon the addition of H_2_O_2_ at ultralow concentrations. When the Co@Fe_3_O_4_ nanozymes with enhanced peroxidase-like activities are specifically located in the acidic microenvironment of the lysosomes, they induce apoptosis of human renal tumor cells (A-498) by catalyzing the decomposition of H_2_O_2_ to generate an ROS burst. Importantly, the Co@Fe_3_O_4_ nanozymes exhibited excellent antitumor activities both *in vitro* and *in vivo* for kidney tumor catalytic therapy.

## Conflicts of interest

There are no conflicts to declare.

## Supplementary Material

RA-009-C8RA05487H-s001
