# Supplementary material for: A cobalt-doped iron oxide nanozyme as a highly active peroxidase for renal tumor catalytic therapy
Source: RSC Adv. 2019 Jun 17;9(33):18815–22. doi: 10.1039/c8ra05487h (PMC9066162; doi:10.1039/c8ra05487h)
Supplement: RA-009-C8RA05487H-s001 [file RA-009-C8RA05487H-s001.pdf]

Electronic Supplementary Material (ESI) for RSC Advances.
This journal is © The Royal Society of Chemistry 2019

Supplementary Information

Cobalt doped iron oxide nanozyme as a highly active peroxidase for renal tumor catalytic therapy

Yixuan Wang[1], Hongjun Li[2*], Lihua Guo[1], Qi Jiang[1], Feng Liu[1]

1. Department of Nephrology, China-Japan Union Hospital of Jilin University, Changchun, 130033, China.

2. The Examination Center, China-Japan Union Hospital of Jilin University, Changchun, 130033, China.

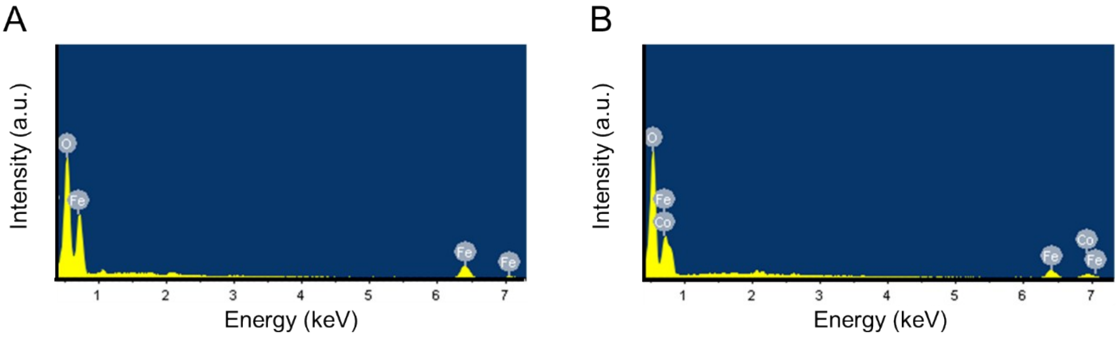

Figure S1 EDX spectrum of the Fe_3O_4 (A) and Co@ Fe_3O_4 (B) nanozymes.

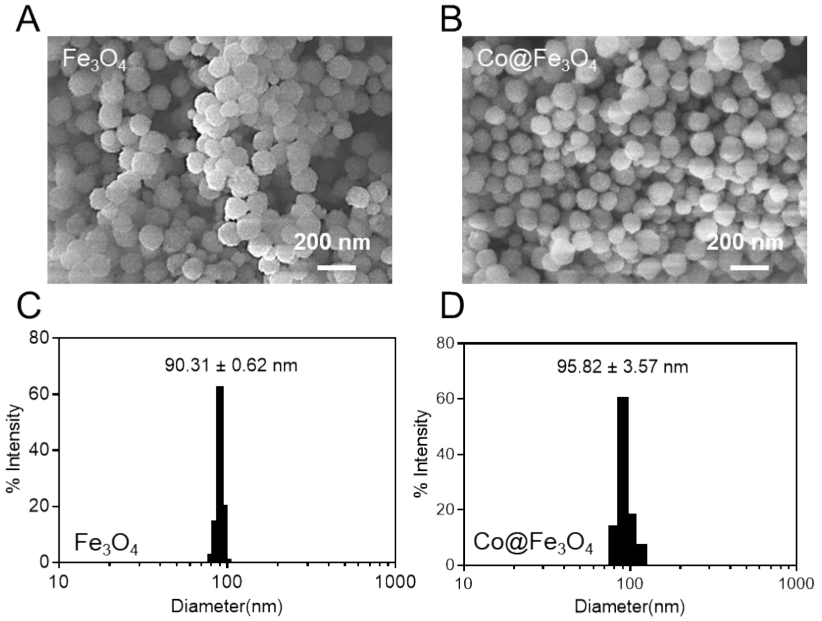

Figure S2 Scanning electron microscope (SEM) and Dynamic light scattering (DLS) analysis of Fe_3O_4 (A, C) and Co@Fe_3O_4 (B, D) nanozymes.

Table S1 Elemental quantification of Co@ Fe_3O_4 by XPS spectra.

Element	Wt %	At %
O	23.26	50.29
Fe	49.07	33.48
Co	27.67	16.23

Table S2 Comparison of the apparent Michaelis-Menton constant (K_M) and maximum initial reaction rate (V_{max}) of the Co@Fe_3O_4 nanozyme with other Fe_3O_4 based nanozymes.

Fe_3O_4 based nanozyme and size (diameter)	Substrate	K_M (mM)	Vmax (M s^{-1})	References
Fe_3O_4, 300 nm	H_2O_2	154	$9.78\times10\text{-}^{8}$	1
	TMB	0.098	$3.44x10^{-8}$	
Fe_3O_4, 13 ± 3.5 nm	H_2O_2	54.6	1.8×10^{-8}	2
	TMB	0.374	2.6×10^{-8}	
GO-Fe_3O_4	H_2O_2	0.71	5.31×10^{-8}	3
	TMB	0.43	13.08×10^{-8}	
Fe_3O_4@Pt	H_2O_2	702.6	7.136×10^{-7}	4

	TMB	0.147	0.711×10^{-7}	
Fe_3O_4@Carbon, 120 nm	H_2O_2	0.38	73.99×10^{-8}	5
	TMB	0.072	17.99×10^{-8}	
Magnetosome	H_2O_2	170.65	9.33×10^{-9}	6
	TMB	0.90	4.45×10^{-9}	
Fe_3O_4@Cu@Cu_2O, 50 nm	H_2O_2	2.3	11.9×10^{-8}	7
	OPDA	0.85	13.2×10^{-8}	
$Mn_{0.5}Fe_{0.5}Fe_2O_4$, 10-11nm	H_2O_2	310	3.63×10^{-6}	8
	TMB	0.139	4.5×10^{-6}	
PB-γ-Fe_2O_3, 9.8 nm	H_2O_2	323.6	1.17×0^{-6}	9
	TMB	0.307	1.06×10^{-6}	
PB-Fe_2O_3, 46 nm	H_2O_2	0.015×10^{-3}	2.28×10^{-7}	10
	TMB	9.95×10^{-3}	1.23×10^{-7}	
PB-Fe_2O_3	H_2O_2	91.54	8.308×0^{-8}	11
	3,5-DTBC	1.22	4.431×0^{-8}	
γ-Fe_2O_3, 122.4 nm	H_2O_2	21.14	1.319×10^{-9}	12
	TMB	0.1709	2.647×10^{-9}	
γ-Fe_2O_3, 20-50 nm	H_2O_2	157.19	1.284×10^{-8}	13
	TMB	0.0887	0.97×10^{-8}	
GO-Fe_2O_3	H_2O_2	305	1.01×10^{-7}	14
	TMB	0.118	5.38×10^{-8}	
Pd@γ-Fe_2O_3	H_2O_2	0.254	1.28×10^{-7}	15
	ABTS	0.049	1.02×10^{-8}	
Co@Fe_3O_4, 95 nm	H_2O_2	0.19	71.5×10^{-8}	This study
	TMB	1.17	37.9×10^{-8}	

References：

1. L. Gao, J. Zhuang, L. Nie, J. Zhang, Y. Zhang, N. Gu, T. Wang, J. Feng, D. Yang, S. Perrett and X. Yan, *Nat Nanotechnol*, 2007, **2**, 577-583.
2. N. V. Vallabani, A. S. Karakoti and S. Singh, *Colloids Surf B Biointerfaces*, 2017, **153**, 52-60.
3. Y. L. Dong, H. G. Zhang, Z. U. Rahman, L. Su, X. J. Chen, J. Hu and X. G. Chen, *Nanoscale*, 2012, **4**, 3969-3976.
4. M. Ma, J. Xie, Y. Zhang, Z. Chen and N. Gu, *Mater Lett*, 2013, **105**, 36-39.
5. Q. An, C. Sun, D. Li, K. Xu, J. Guo and C. Wang, *Acs Appl Mater Inter*, 2013, **5**, 13248-13257.
6. K. Li, C. Chen, C. Chen, Y. Wang, Z. Wei, W. Pan and T. Song, *Enzyme Microb Tech*, 2015, **72**, 72-78.
7. Z. Wang, M. Chen, J. Shu and Y. Li, *J Alloy Compd*, 2016, **682**, 432-440.
8. D. Bhattacharya, A. Baksi, I. Banerjee, R. Ananthakrishnan, T. K. Maiti and P. Pramanik, *Talanta*, 2011, **86**, 337-348.
9. X.-Q. Zhang, S.-W. Gong, Y. Zhang, T. Yang, C.-Y. Wang and N. Gu, *J Mater Chem*, 2010, **20**, 5110-5116.
10. A. K. Dutta, S. K. Maji, D. N. Srivastava, A. Mondal, P. Biswas, P. Paul and B. Adhikary,

Journal of Molecular Catalysis A: Chemical, 2012, **360**, 71-77.

11. A. K. Dutta, S. K. Maji, P. Biswas and B. Adhikary, *Sensors and Actuators B: Chemical*, 2013, **177**, 676-683.
12. Q. Liu, L. Zhang, H. Li, Q. Jia, Y. Jiang, Y. Yang and R. Zhu, *Materials Science and Engineering: C*, 2015, **55**, 193-200.
13. A. Roy, R. Sahoo, C. Ray, S. Dutta and T. Pal, *RSC Advances*, 2016, **6**, 32308-32318.
14. L. Song, C. Huang, W. Zhang, M. Ma, Z. Chen, N. Gu and Y. Zhang, *Colloids and Surfaces A: Physicochemical and Engineering Aspects*, 2016, **506**, 747-755.
15. M. Kluenker, M. Nawaz Tahir, R. Ragg, K. Korschelt, P. Simon, T. E. Gorelik, B. Barton, S. I. Shylin, M. Panthöfer, J. Herzberger, H. Frey, V. Ksenofontov, A. Möller, U. Kolb, J. Grin and W. Tremel, *Chem Mater*, 2017, **29**, 1134-1146.
